# Cross-species Comparison of Proteome Turnover Kinetics*[Fn FN2]

**DOI:** 10.1074/mcp.RA117.000574

**Published:** 2018-01-10

**Authors:** Kyle Swovick, Kevin A. Welle, Jennifer R. Hryhorenko, Andrei Seluanov, Vera Gorbunova, Sina Ghaemmaghami

**Affiliations:** From the ‡Department of Biology, University of Rochester, NY;; §University of Rochester Mass Spectrometry Resource Laboratory, NY

## Abstract

The constitutive process of protein turnover plays a key role in maintaining cellular homeostasis. Recent technological advances in mass spectrometry have enabled the measurement of protein turnover kinetics across the proteome. However, it is not known if turnover kinetics of individual proteins are highly conserved or if they have evolved to meet the physiological demands of individual species. Here, we conducted systematic analyses of proteome turnover kinetics in primary dermal fibroblasts isolated from eight different rodent species. Our results highlighted two trends in the variability of proteome turnover kinetics across species. First, we observed a decrease in cross-species correlation of protein degradation rates as a function of evolutionary distance. Second, we observed a negative correlation between global protein turnover rates and maximum lifespan of the species. We propose that by reducing the energetic demands of continuous protein turnover, long-lived species may have evolved to lessen the generation of reactive oxygen species and the corresponding oxidative damage over their extended lifespans.

Within a cell, proteins are in a state of flux and are continually degraded and re-synthesized (1). The process of protein turnover plays a critical quality control function in cells. Over time, proteins tend to become damaged by a number of stochastic mechanisms including oxidation, nitrosylation, and aggregation (2). The process of turnover ensures that damaged proteins are perpetually replaced by a nascent pool of undamaged, functional proteins. Additionally, protein turnover plays an important role in the regulation of protein expression levels. The constant turnover of proteins allows their steady-state levels to adjust in response to changes in synthesis rates (3, 4).

Recent advances in quantitative proteomics and mass spectrometry have enabled the measurement of protein turnover kinetics on proteome-wide scales (5–10). These studies have shown that turnover rates are highly variable within the proteome, with protein half-lives ranging from minutes to years. Several factors can influence the turnover rates of proteins *in vivo*. In some proteins, identities of N-terminal residues appear to have a strong influence on half-lives, a phenomenon referred to as the “N-end rule” (11). The presence of longer sequence domains, termed “degrons,” have also been shown to affect protein turnover. For example, sequences rich in proline, glutamic acid, serine, and threonine have been shown to act as robust degradative markers (12, 13). In addition to sequence determinants, physical properties of proteins such as isoelectric points, surface areas, thermodynamic stabilities and molecular weights can influence half-lives (14–16). However, none of these determinants are universally applicable to the entirety of the proteome and it is currently not possible to predict the half-life of a protein based solely on its sequence and structure.

The turnover rate of a protein is not only dependent on its sequence and structure, but also on the relative activity and selectivity of proteolytic pathways within the cell. Hence, proteins of identical sequence can have vastly different half-lives within different cell types, tissues and environmental conditions (6, 7). Within a cell, proteins can be degraded by several proteolytic pathways and proteases. In eukaryotes, the two major degradation pathways with broad selectivity are autophagy and the ubiquitin proteasome system (UPS)^1^ (17). These two pathways are believed to have distinct functions in maintaining protein homeostasis. Autophagy has been shown to be involved in the degradation of damaged organelles, protein aggregates, long-lived proteins, recycling of macromolecules during periods of starvation and large protein complexes. In comparison, the UPS is believed to be primarily responsible for the degradation of short-lived and regulatory proteins and rapid elimination of damaged proteins (18, 19). As the primary catabolic mechanisms of eukaryotic proteomes, the relative activities of autophagy and UPS can play a major role in establishing the half-lives of intracellular proteins *in vivo*.

To what extent are the turnover kinetics of individual proteins conserved across species? Although a few proteomic studies have addressed this question in model organisms, the results have been somewhat contradictory. For example, a comparison of yeasts *Saccharomyces cerevisiae* and *Schizosacharomyces pombe* observed very little conservation in protein turnover rates between the two species (20). Conversely, an analysis of two immortal cell lines, HeLa and C212 myoblasts, originating from human and mouse tissues respectively, indicated a somewhat higher correlation in protein turnover rates (8). In another study, a comparison of *in vivo* turnover rates in two rodents, mouse and vole, measured in two separate studies, also showed limited correlation (21). However, to date, a systematic cross-species comparison of protein turnover rates among a set of organisms has not been conducted in a single standardized study. Here, we have used dynamic isotopic labeling and quantitative proteomics to globally quantify protein turnover kinetics in primary dermal fibroblasts isolated from eight different rodent species. The species were chosen to represent a range of evolutionary distance and physiological properties, including body mass, metabolic rate, and lifespan. The results provide a systematic comparison of proteome turnover kinetics in a single cell type across multiple species.

## EXPERIMENTAL PROCEDURES

### 

#### 

##### Experimental Design and Statistical Rationale

**Fig. 1.**
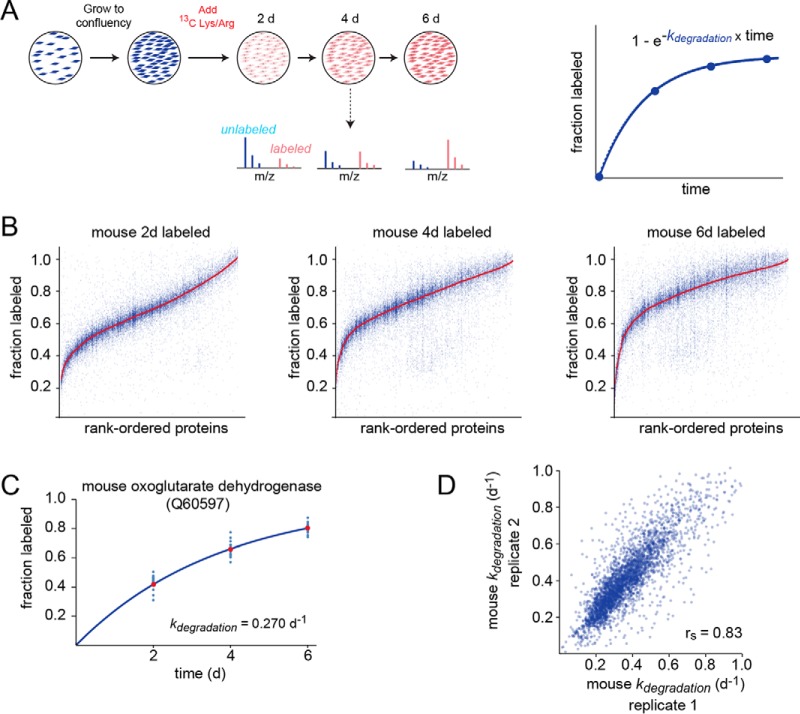
**Quantitation of *k_degradation_* in rodent fibroblasts.** *A*, Experimental design. Blue and red colors indicate unlabeled and isotopically labeled spectra/cells, respectively. The plot shows theoretical labeling kinetic data for a peptide fitted to a first order exponential equation for determination of *k_degradation_*, based on the model described in the Experimental Procedures. *B*, Rank-size distribution plots showing the fractional labeling of peptides matched to each protein within the mouse proteome at different time-points. Vertical columns of blue points on the plot represent data for peptides matched to specific proteins. Red points indicate the median value for peptides matched to a given protein. Note that the range of measured fractional labeling for peptides within each protein is significantly narrower than the entire range of all measured peptides within the proteome. *C*, Labeling kinetics of oxoglutarate dehydrogenase, shown as an example of protein-level determination of *k_degradation_* values. Blue dots indicate all peptides mapped to the protein and red dots indicated the median of all peptides. The line is a fit to the exponential equation shown in (*A*). *D*, Biological replicate measurements of *k_degradation_* in mouse, indicating the precision of experiments.

The theoretical rationale for the assay workflow (Fig. 1*A*) is discussed in the Results section. For every kinetic experiment, 4 cultures of dermal fibroblasts isolated from different rodents were grown to confluency and labeled for varying times (0d, 2d, 4d, and 6d) in SILAC media. The fractional labeling of the peptides were analyzed at the MS1 level using the procedures described below. In a second biological replicate, the experiment with mouse cells was repeated in order to assess the experimental variability. The determination of the rate constants for fractional labeling was conducted by least squares regression analysis of the time-resolved data fitted to equations derived from the kinetic model described below and the goodness of fit was assessed by R^2^ values. The spread of rate constants for peptides encompassing the same protein was assessed by measuring the coefficient of variation. The correlation of measured rates between species was mostly assessed by conducting pairwise comparisons. For this analysis, Spearman rank correlation was used as the measured degradation rates were not normally distributed (see below).

##### Cell Culture and Stable Isotope Labeling

All dermal fibroblasts were isolated and cultured according to the protocols described by Seluanov *et al.* (22, 23). The isolated fibroblasts were grown in EMEM media supplemented with 15% fetal bovine serum (FBS), 100 U/ml penicillin, and 100 U/ml streptomycin and cultured. Before isotopic labeling, cultures were grown to 100% confluency. The sole exceptions were cultures from naked mole rats that ceased division at ∼70% confluency because of the phenomenon of early-contact inhibition (23). Once cells ceased cell division because of contact inhibition, they were maintained in a quiescent state for 4 days. Subsequently, the cells were acclimated to the labeling media (EMEM supplemented with 15% dialyzed FBS (Thermo Scientific, Waltham, MA), 100 U/ml penicillin, and 100 U/ml streptomycin) for 4 days before labeling. After four additional days in adaptation media, the cultures were introduced to MEM media for SILAC (Thermo Scientific) supplemented with l-arginine:HCl (^13^C6, 99%) and l-lysine:2HCl (^13^C6, 99%; Cambridge Isotope Laboratories, Tewksbury, MA) at concentrations of 0.13 g/l and 0.0904 g/l respectively, 15% dialyzed FBS, 100 U/ml penicillin, and 100 U/ml streptomycin. After 0, 2, 4, and 6 days of labeling, cells were harvested, washed with PBS, and pellets were frozen before further analysis. To evaluate the precision of our measurements, biological replicate experiments were conducted for mouse cells from two independent cultures.

##### Mass Spectrometry Sample Preparation

Cells were lysed in a buffer containing 8 m Urea, 150 mm NaCl, and 50 mm HEPES (pH = 9.0). Cell pellets were re-suspended in 50 μl of lysis buffer per 10^6^ cells and subjected three times to sonication using a high-energy sonicator (QSonica, Newtown, CT) for 10s with 60s resting periods on ice. The samples were centrifuged for 5 min at 16,000 × *g* and the supernatants were then transferred to new Eppendorf tubes. Protein concentrations were measured by the bicinchoninic assay (BCA) kit (Thermo Scientific). Subsequent experiments were performed using 50 μg of total protein from each culture. Reduction of disulfide bonds was performed with 5 mm Tris(2-carboxyethyl)phosphine (TCEP) Bond-breaker (Thermo Scientific) at RT for 1 h, and protein alkylation was performed with 10 mm iodoacetamide (IAA) at RT for 30 min in darkness. DTT was added to 1 mm to quench IAA and samples were diluted to a urea concentration of less than 1 m with 50 mm HEPES. To derive tryptic peptides, 1 μg of trypsin (selective cleavage on the C-terminal side of lysine and arginine residues) was added and the samples were incubated overnight at 37 °C. To quench trypsin, formic acid was added to a final concentration of 1%.

To increase proteome coverage, high-pH fractionation was conducted on extracts before LC-MS/MS using the Pierce High-pH Reverse-Phase Peptide Fraction Kit (Thermo Scientific). Eight different elution buffers were made in 0.1% trimethylamine with 5%, 7.5%, 10%, 12.5%, 15%, 17.5%, 20%, and 50% acetonitrile added. After conditioning the column with acetonitrile and 0.1% trifluoroacetic acid (TFA), the samples were added and centrifuged. Alternatively, for some of the samples (mouse replicates), extracts were fractionated using homemade C18 spin columns. Eight different elution buffers were made in 100 mm ammonium formate (pH 10) with 5%, 7.5%, 10%, 12.5%, 15%, 17.5%, 20%, and 50% acetonitrile added. After conditioning the column with acetonitrile and 100 mm ammonium formate, the samples were added and centrifuged. An ammonium formate wash was performed to remove any residual salt before the eight elutions were collected in fresh tubes. All fractions were then dried down and re-suspended in 20 μl of 0.1% TFA.

##### LC-MS/MS Analysis

Peptide extracts were injected onto homemade C18 columns with 1.8 μm beads (Sepax, Newark, DE), using an Easy nLC-1000 HPLC system (Thermo Fisher) connected to a Q Exactive Plus mass spectrometer (Thermo Fisher). For the elution gradient, 0.1% formic acid in water was used as Solvent A and 0.1% formic acid in acetonitrile was used as Solvent B. For samples that were pre-fractionated with Pierce High pH fractionation columns, the gradient began at 3% B and held for 2 min, increased to 8% B over 5 min, then increased to 30% B over 68 min, until finally increasing to 70% B over 3 min. The gradient was held at 70% B for 3 min until returning to 0% B over 2 min, then was held there for 8 min to re-equilibrate the column. For samples that were pre-fractionated with homemade C18 spin columns, optimized LC gradients were used for each fraction. For fractions 1 and 2, the gradient went from 2–20% B over 71 min. For fractions 3 and 4, the gradient went from 3–30% B over 71 min. For fractions 5 and 6, the gradient went from 8–30% B in 71 min, and for fractions 7 and 8 the gradient went from 11–35% B in 71 min. For all methods, the gradient was ramped up to 70% B after the peptide separation and was held there for 3 min, and then returned to 0% B over 2 min and held there for 8 min to re-equilibrate the column. The total run time for all methods was 90 min.

The Q Exactive Plus was operated in data-dependent mode, with a full MS1 scan followed by 20 data-dependent MS2 scans. The full scan was done over a range of 400–1400 *m*/*z*, with a resolution of 70,000 at *m*/*z* of 200, an AGC target of 1e6, and a maximum injection time of 50 ms. The MS2 scans were performed at 17,500 resolution, with an AGC target of 5e4 and a maximum injection time of 55 ms. The isolation width was 1.5 *m*/*z*, with an offset of 0.3 *m*/*z*, and a normalized collision energy of 27.

##### Data Analysis

MS2 data for all samples were searched against the *M. musculus* (22,305 entries, downloaded 8/7/2017), *R. norvegicus* (21,369 entries, downloaded 10/18/2017), *M. auratus* (20,192 entries, downloaded 10/24/2017), *C. porcellus* (18,757 entries, downloaded 10/18/2017) or *H. Glaber* (21,436 entries, downloaded 10/25/2017) uniprot databases using the integrated Andromeda search engine with MaxQuant software (24). The maximum allowable numbers of missed cleavages in all searches were two and the FDR thresholds were set to a maximum of 1%. SILAC peptide and protein quantification were performed with MaxQuant using the parameter settings listed in Table S1. For each peptide, heavy to light (H/L) SILAC ratio was determined by MaxQuant using a model fitted to all isotopic peaks within all scans that corresponding peptide spectral matches were detected (24). The H/L ratio for each peptide, obtained MaxQuant outputs, was subsequently converted to fraction labeled (H/(H+L)) measurements.

The determination of degradation rate constants (*k_degradation_*) from fraction labeled measurements were conducted in accordance to the kinetic model outlined in the next section. To obtain *k_degradation_* measurements for each peptide, plots of fraction labeled as a function of time were fitted to a single exponential function (equation 8, below) using least squares fitting. To determine *k_degradation_* at the protein level, fraction labeled measurements for all peptides and time-points mapped to specific proteins were combined in single aggregated kinetic plots and fitted to a single exponential function using the least squares method to determine *k_degradation_*. All reported *k_degradation_* measurements at the protein level were required to pass two quality control criteria: 1) at least two unique peptide sequences were quantified in one or more time-points and 2) at least one peptide was quantified in two or more time-points.

##### Kinetic Model

The kinetic model applied in this study has been described previously (6, 25, 26) and is based on the following assumptions:

1. Protein synthesis is a zero order process with respect to protein concentration.
2. Protein degradation occurs at a constant fractional rate that is uniform for the entire protein pool. Thus, protein degradation can be modeled as a first order process with respect to protein concentration.
3. The total protein concentration of each cell does not change during the experimental time-course and the system is at steady-state.

Based on these assumptions, we can devise the following rate equations for clearance of unlabeled proteins and accumulation of labeled proteins:

(1) $$\frac{d\left[unlabeled\;protein\right]}{dt}=-\left(k_{deg}+k_{div}\right)\left[unlabeled\;protein\right]$$

(2) $$\frac{d\left[labeled\;protein\right]}{dt}=k_{syn}-\left(k_{deg}+k_{div}\right)\left[labeled\;protein\right]$$

Where *k_syn_* is the zero-order rate constant for protein synthesis, *k_deg_* is the first-order rate constant for protein degradation and *k_div_* is the first order rate constant for cell division.

We solve for *[unlabeled protein](t)* using the constraint *[unlabeled protein](0)* = *[protein]_steady−state_* and for *[labeled protein](t)* using the constraint *[labeled protein](0)* = *0.*

(3) $$\left[unlabeled\;protein\right]\left(t\right)=\left[protein_{steady-state}\right]e^{-\left(k_{deg}+k_{div}\right)\times \text{t}}$$

(4) $$\left[labeled\;protein\right]\left(t\right)=\left[protein_{steady-state}\right]-\left[protein_{steady-state}\right]e^{-\left(k_{deg}+k_{div}\right)\times \text{t}}$$

Where

(5) $$\left[protein_{steady-state}\right]=\frac{k_{syn}}{\left(k_{deg}+k_{div}\right)}$$

Because our protein labeling measurements are fractional (*i.e.* internally normalized with respect to total steady-state protein levels), the observed fractional labeling is derived as:

(6) $$fraction\;labeled\;protein\left(t\right)=\left[labeled\;protein\right]\left(t\right)/\left[protein_{steady-state}\right]=1-e^{-\left(k_{deg}+k_{div}\right)\times \text{t}}$$

All experiments were conducted in quiescent cells, where the rate of cell division is zero. Hence, equations 5 and 6 simplify to:

(7) $$quiescent\left[protein_{steady-state}\right]=\frac{k_{syn}}{k_{deg}}$$

(8) $$quiescent\;fraction\;labeled\;protein\left(t\right)=1-e^{-k_{deg}\times t}$$

As a first order rate constant, the relationship between *k_deg_* and half-life of a protein is as follows:

(9) $$half\text{-}life=\log2/k_{deg}$$

## RESULTS

### 

#### 

##### Experimental Design and Validation of Data Precision

To conduct a global cross-species comparison of protein turnover kinetics, skin fibroblasts were isolated from eight different rodent species: mouse, rat, hamster, guinea pig, beaver, chinchilla, blind mole rat, and naked mole rat (22, 27) (Table I). The species were chosen to represent a range of organismal properties, including evolutionary distance, body mass, metabolic rate, and lifespan. The rodents belong to three distinct suborders within the order *Rodentia* (*Myomorpha*, *Castorimorpha* and *Hystricomorpha*) and are separated by ∼73 million years of evolution.

**Table I:** Rodents analyzed in this study. The lifespan and body mass data were obtained from AnAge database (45)

		adult body mass (g)	maximum lifespan (yr)
*Myomorpha*	mouse *(Mus musculus)*	30	4
	rat *(Rattus norvegicus)*	400	5
	hamster *(Mesocricetus auratus)*	105	4
	blind mole rat *(Nannospalax ehrenbergi)*	160	20
*Castorimorpha*	beaver *(Castor canadensis)*	20,000	24
*Hystricomorpha*	chinchilla *(Chincilla lanigera)*	642	17
	guinea pig *(Cavia porcellus)*	728	12
	naked mole rat *(Hetercephalus glaber)*	35	32

The experimental design is illustrated in Fig. 1*A*. Before performing dynamic SILAC experiments, cultures were grown to a contact-inhibited quiescent state. By conducting the analyses in a non-dividing state, we ensured that cellular proliferation was not contributing to the kinetics of isotopic labeling. In contrast, when isotopic labeling experiments are conducted in dividing cells, the fractional rate of labeling is the summation of protein degradation and cellular proliferation rates (7). Thus, in dividing cells, the kinetics of protein degradation typically can not be measured for proteins whose degradation is significantly slower than the doubling time of the cell (28). Here, by maintaining cells in a quiescent state throughout the course of labeling, we were able to analyze the turnover kinetics of long-lived proteins whose half-lives are significantly longer than the rate of cellular proliferation.

The fractional labeling of tryptic peptides were measured after 0, 2, 4, and 6 days of SILAC labeling. The data were analyzed in the context of the kinetic model outlined under Experimental Procedures. The kinetics of fractional labeling were fit to a single exponential equation and the first order rate constants for degradation (*k_degradation_*) and the corresponding half-lives were measured for each peptide. In the following discussion, the terms *k_degradation_* and “turnover rate” will be used synonymously.

Different tryptic peptides mapped to the same protein provide independent probes for measurement of its turnover rate. As expected, we observed that peptides mapped to the same protein generally had similar measured turnover rates (Fig. 1*B*). This is evidenced by the fact that the range of coefficients of variation (CV) values for peptide *k_degradation_* measurements within a protein is low in comparison to the CV of all analyzed peptide *k_degradation_* measurements within the proteome (supplemental Fig. S1). To measure *k_degradation_* at the protein level, data for all peptides mapped to a given protein were aggregated and fit with a single exponential equation (Fig. 1*C*). To assess the level of experimental error in the measurements, we analyzed mouse cells in two biological replicates. The results indicate a high level of correlation among biological replicates (Fig. 1*D*), validating the overall precision of the proteomic data.

##### Distribution of k_degradation_ Measurements

**Fig. 2.**
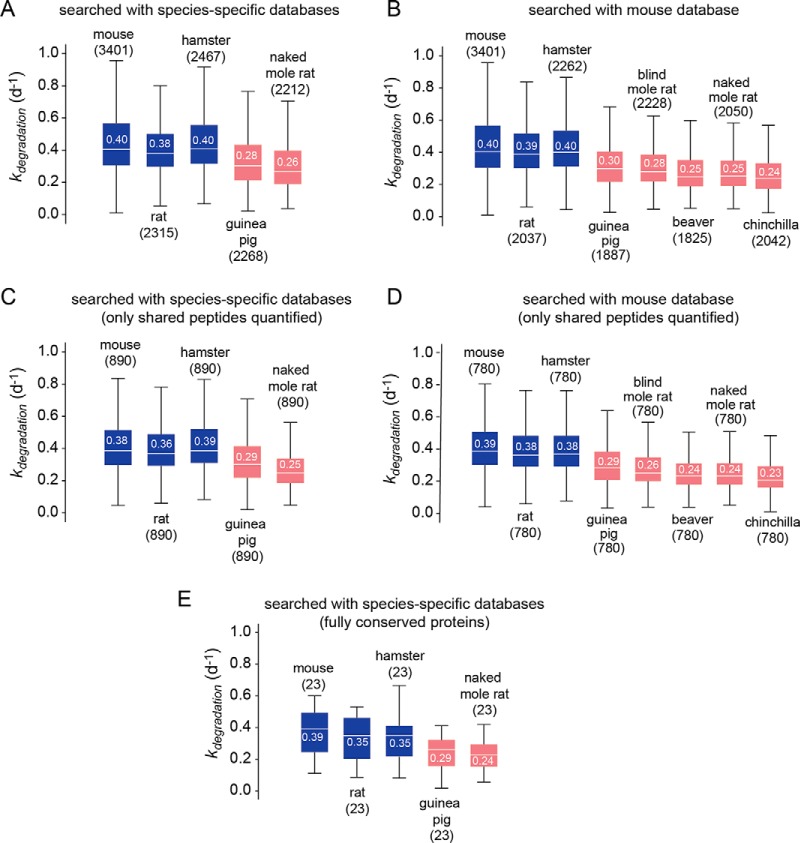
**The distribution of *k_degradation_* measurements in fibroblasts derived from different rodents.** Box plots indicate the distribution of protein *k_degradation_* values for different species. White lines and numbers indicate the median, boxes indicate the interquartile range, and the brackets indicate the entire range, excluding far outliers (>2*S.D.). Numbers in parentheses indicate the number of proteins analyzed in each box plot. The data were either searched with species-specific sequence database (*A*, *C*, *E*) or mouse sequence database (*B*, *D*). Plots (*C*) and (*D*) are limited to measurements based on quantification of peptides shared among the species analyzed in each plot. Plot E is limited to proteins whose sequences are fully conserved among the indicated species. In each plot, the median of distributions indicated in blue significantly differ from those indicated in red with a *p* value of less than 1e-10 (*A–D*) or 1e-3 (*E*) using the Mann Whitney *U* test.

Using the procedure outlined above, we globally measured turnover rates in fibroblasts isolated from the eight rodent species (Table II, Fig. 2). For species with well-annotated proteomes (mouse, rat, hamster, guinea pig and naked mole rat), the MS/MS data were searched against species-specific uniprot databases (Fig. 2*A*, 2*C*, 2*E*). Additionally, in order to extend our analysis to non-model organisms for which well-annotated sequence databases were not available, we searched the MS/MS data from all rodent species against the mouse uniprot database (Fig. 2*B*, 2*D*). The latter approach necessarily reduces the coverage of proteomic data for non-mouse rodents. However, searching data from all rodents against the same well-annotated sequence database avoids potential errors in false assignment of protein orthology when conducting inter-species comparisons of protein *k_degradations_* measurements.

**Table II:** Coverage of dynamic proteomic experiments. For a given species, the MS/MS data were searched against either mouse or the species-specific database as indicated. “PSM” indicates the number of peptide spectral matches. “Unique peptides” indicates the number of unique peptide sequences. “Protein Groups” indicates the number of homologous protein groups. The reported number of quantified Protein Groups is limited to those for which heavy to light ratios (H/L) could be quantified by two peptide spectra. The reported number of k_degradation_ values measured for Protein Groups is limited to those where two distinct peptides could be measured in two or more time-points

Experiment	Searched database	Time-point	Detected			H/L quantified		*k_degradation_* analyzed	
			PSMs	Unique peptides	Protein groups	Unique peptides	Protein groups^a^	Unique peptides	Protein groups^b^
Mouse	Mouse	2d	93418	38449	5350	25662	3086		
		4d	105193	43803	5165	26680	2775	41682	3401
		6d	94460	42098	5130	24582	2676		
Rat	Mouse	2d	85626	25290	3727	18213	2243		
		4d	55709	20527	3517	12422	1682	22713	2037
		6d	55589	18695	2931	11366	1470		
Hamster	Mouse	2d	90671	25932	4122	17870	2339		
		4d	55242	19486	3430	12057	1732	21795	2262
		6d	75347	24755	4003	14487	2013		
Guinea Pig	Mouse	2d	56881	16712	2916	12438	1845		
		4d	54505	16368	2888	11317	1690	16169	1887
		6d	47263	14240	2714	9869	1516		
Blind Mole Rat	Mouse	2d	80676	21472	3575	15618	2190		
		4d	62582	19524	3380	13385	1880	20230	2228
		6d	80652	22379	3835	13783	1939		
Chinchilla	Mouse	2d	54698	16152	3104	12189	1983		
		4d	51188	15153	3027	11538	1908	15975	2042
		6d	49061	14499	2962	10907	1857		
Beaver	Mouse	2d	51467	16031	2698	11810	1721		
		4d	44979	14758	2602	11116	1638	15498	1825
		6d	42644	13874	2554	10307	1528		
Naked Mole Rat	Mouse	2d	63613	20150	3194	14736	1979		
		4d	54968	17538	3212	11297	1667	20246	2050
		6d	59707	20679	3380	12770	1674		
Rat	Rat	2d	114368	34682	4345	23925	2633		
		4d	73573	27353	3988	15834	1957	29902	2315
		6d	76736	26326	3525	15148	1763		
Hamster	Hamster	2d	122553	36434	5068	24277	2906		
		4d	78198	28174	4309	16319	2117	29887	2467
		6d	104367	35594	4973	19510	2492		
Guinea Pig	Guinea Pig	2d	100119	30356	3908	21732	2583		
		4d	95432	29581	3952	19660	2350	28732	2268
		6d	88322	27635	3823	17898	2210		
Naked Mole Rat	Naked Mole Rat	2d	111471	37261	4665	25724	2875		
		4d	95305	31719	4507	19134	2337	33051	2212
		6d	45754	16739	3267	9345	1531		

^a^ Based on at least two quantified peptides.

^b^ Limited to proteins for which at least two unique peptide sequences were quantified at two or more time-points.

The coverages of the proteomic experiments are tabulated in Table II. For analysis of *k_degradations_* values, we only considered proteins for which two or more unique peptides were quantified at two or more time-points (see Experimental Procedures). The complete search results and quantitation datasets are included in Supplementary Information (supplemental Tables S2–S5), and the raw data are publicly available in the PRIDE database (www.ebi.ac.uk/pride/ accession #: PXD007598).

The distribution and median of *k_degradation_* measurements, determined by searches against species-specific and mouse databases, are shown in Figs. 2*A* and 2*B*, respectively. The measured rates spanned approximately one order of magnitude from ∼0.1 to ∼1.0 d^−1^, corresponding to half-lives of ∼15 h to ∼1 week. For four of the rodent species (rat, hamster, guinea pig and naked mole rat), the data could be searched against both mouse and species-specific databases. For these species, searching against the mouse database reduced the protein coverage by ∼8–15% but did not significantly alter the distribution of *k_degradations_* measurements (comparison of Figs. 2*A* and 2*B*). We conclude that the usage of the mouse database to search data from other rodent species does not significantly bias the observed global distribution of *k_degradation_* (as would be expected if the dataset was being significantly enriched for highly conserved proteins). This is largely because the majority of orthologous proteins present in these species (and not only highly conserved proteins) share at least two tryptic peptides of identical sequence.

##### Inter-species Variability in k_degradation_ Distributions

The data indicate that there are significant differences in *k_degradation_* distributions among some of the rodent species. To determine if these variations were due to differences in the composition of the peptide sequences quantified in each species, we limited the analysis to peptide sequences that were shared among all species (Fig. 2*C*, 2*D*). The results indicate that differences in *k_degradation_* distributions are observable even when the analysis is limited to tryptic peptides shared among species.

We reasoned that there may be two general mechanisms for inter-species variability in protein turnover kinetics. First, protein targets may have diverged in sequence during the course of evolution, altering their relative susceptibility to cellular degradation pathways. Second, the relative activities of cellular degradation pathways (*e.g.* autophagy and UPS) may be variable among cells obtained from different species. To distinguish between these two possibilities, we identified a set of fully conserved proteins with identical amino acid sequences among four of the species with well annotated protein sequence databases (mouse, rat, hamster, guinea pig and naked mole rat) (supplemental Table S6). The data indicate that the inter-species variation in k_degradation_ distributions observed for the proteome at large is also evident for fully conserved proteins (Fig. 2*E*). The results suggest that systematic shifts in turnover kinetics among species are largely driven by differences in activities of the cellular degradation machinery rather than divergence in sequences of protein targets.

##### Correlation of k_degradation_ and Lifespan

**Fig. 3.**
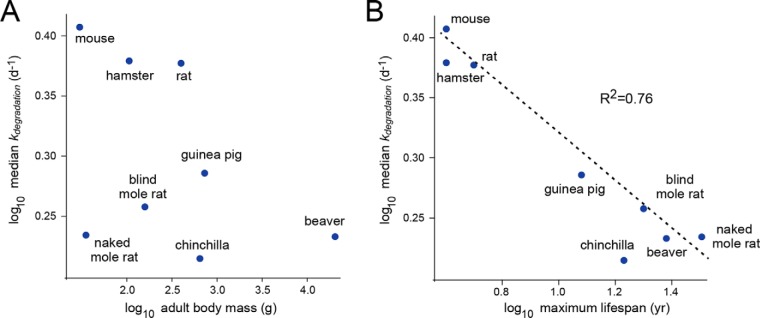
**Median *k_degradation_* values within rodent proteomes negatively correlate with lifespan.** Correlation of median *k_degradation_* values with adult body mass (*A*) and maximal lifespan (*B*) of rodent species.

We calculated median degradation rates within the shared proteome of each species and assessed the correlation of these values to several organismal properties including body mass and lifespan (Fig. 3). The phenotype that showed the strongest correlation to the median degradation rate was the maximal lifespan of the organism. Specifically, we observed a strong negative correlation between these two parameters, indicating that long-lived rodents had slower overall rates of degradation in comparison to short-lived rodents (Fig. 3*B*). After correcting for the effect of phylogenetic distance using the method of independent contrasts (29), the correlation between median degradation rate and the maximal lifespan had a two-tailed *p* value of 0.03 (supplemental Fig. S2). The results suggest that maximal lifespan and protein turnover kinetics are independently correlated.

##### Cross-species Correlations of k_degradation_

**Fig. 4.**
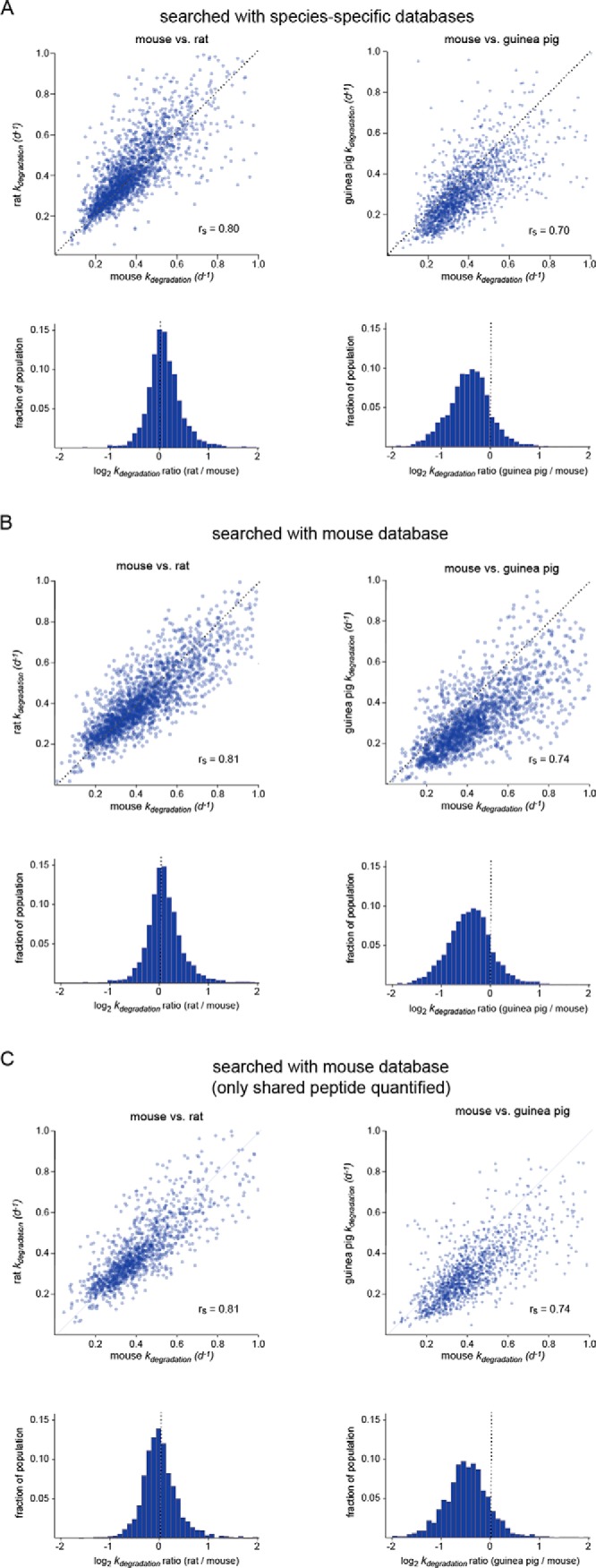
**Cross-species correlations of *k_degradation_* measurements.** Pairwise associations and log_2_ ratios of *k_degradation_* measurements for mouse *versus* rat, and mouse *versus* guinea pig comparisons. Data were generated with searches against species-specific sequence databases (*A*), mouse database (*B*) or limited to peptides shared among the three species (*C*). The dotted lines indicate the identity lines. Spearman rank-order correlation coefficients (r_S_) are indicated. Other cross-species correlations are shown in supplemental Fig. S3.

We next analyzed inter-species correlation and variability in relative *k_degradation_* values by conducting pairwise comparisons of protein measurements among species. As examples, Fig. 4 shows pairwise comparisons of protein turnover kinetics between mouse and rat (two species in the same suborder), and between mouse and guinea pig (two species in different suborders.) The comparisons were used to quantify inter-species correlations in turnover kinetics. For this analysis, we used the nonparametric Spearman's rank-order correlation (r_S_) instead of Pearson product-moment correlation (r) as *k_degradation_* values were generally not normally distributed and some heteroscedasticity was evident in the data, with faster *k_degradation_* values being generally more divergent than slower *k_degradation_* values (for example, see Fig. 4*A*). The analysis indicates that the correlation in protein turnover rates between mouse and rat is greater than the correlation between mouse and guinea pig (r_S_ = 0.80 and 0.70, respectively.) Thus, in the latter comparison, in addition to a systematic shift, there was a decrease in correlation of turnover rates, suggestive of greater stochastic variability in relative degradation rates between the proteome of the two species. The magnitude of this effect was independent of search strategy (using either species-specific or mouse databases) and did not change when the analysis was repeated using only peptides quantified in all three species (mouse, rat and guinea pig) (Fig. 4*B*, 4*C*).

**Fig. 5.**
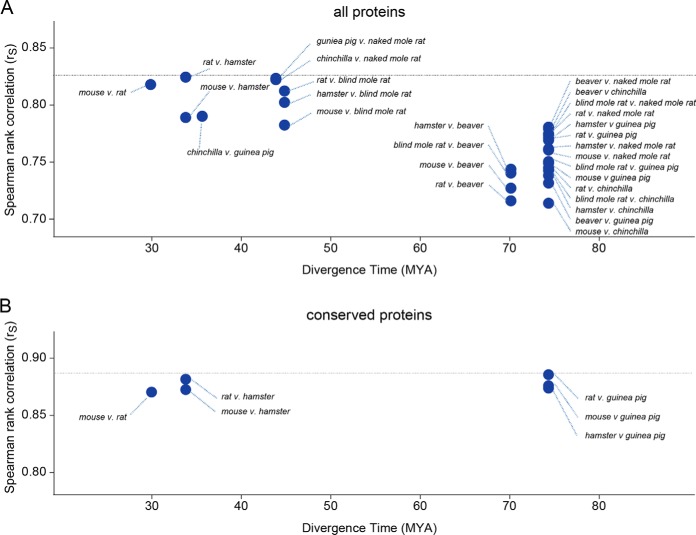
**Spearman rank-order correlation coefficients (r_S_) for cross-species comparisons of *k_degradation_* measurements as a function of evolutionary distance.** *A*, includes data for all proteins, and (*B*) includes data for proteins whose sequences are fully conserved between the two species. Divergence times between pairs of species were obtained from TimeTree (46).

We conducted similar pairwise comparisons of all analyzed rodent species (supplemental Fig. S3). For this analysis, we used data generated by searches against the mouse database and limited each protein-level comparison to peptide sequences shared between each pair of species. Analysis of r_S_ values indicated that the correlation among turnover rates generally decrease as a function of evolutionary distance. This is evidenced by the fact that r_S_ values among species with shorter evolutionary divergence times are generally higher than r_S_ values among species with longer evolutionary divergence times (Fig. 5*A*). For many of the interspecies comparisons with divergence times less than 50 million years, the r_S_ values are very close to the correlation among biological replicates, suggesting that most of the observed variability can be accounted for by experimental error alone. Conversely, for many of the inter-species comparisons with divergence times greater than 50 million years, r_S_ values are significantly lower (*p* value <0.0001). Together, the results suggest that relative protein turnover kinetics have gradually diverged over the course of evolution.

We observed that the inter-species r_S_ values for fully conserved proteins are significantly higher than the proteome at large (Fig. 5*B*). Furthermore, the r_S_ values for fully conserved proteins do not vary as a function of evolutionary distance. These observations are consistent with the idea that variability in relative turnover rates within proteomes of distant species are related to changes in sequences of protein targets (see Discussion below).

##### Relationship Between Protein Function and Conservation of Turnover Kinetics

**Fig. 6.**
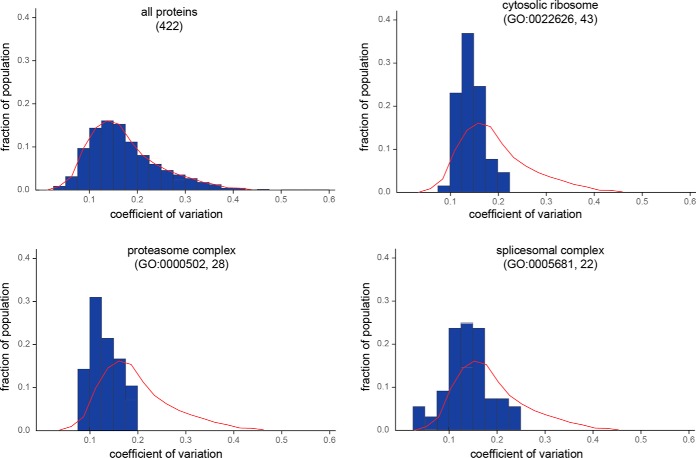
**Variability of protein *k_degradation_* values shared across eight rodent species.** Distribution of coefficient of variations (CV) for *k_degradation_* values for the all proteins, and protein subunits of the ribosome, proteasome and splicesome complexes. The red line outlines the distribution of all proteins for comparison. The data indicate that *k_degradation_* values for the three complexes are significantly less variable than the proteome at large. The gene ontology term and the number of proteins associated with that term in the dataset are indicated in parentheses.

We next investigated whether there is a relationship between protein function and conservation of turnover kinetics across species. We normalized *k_degradation_* measurements relative to the median *k_degradation_* value within each species and measured the coefficient of variation (CV) of relative turnover rates for shared proteins across the eight rodents. Fig. 6 illustrates the distribution of CVs across the shared proteome. We used the GOrilla algorithm (30) to search for gene ontology (GO) terms whose constituents were enriched in the subset of proteins with low *k_degradation_* CV values (supplemental Fig. S4, supplemental Table S7). Among the GO categories with the most highly conserved (low CV) *k_degradation_* values were the ribosome, the proteasome and the splicesome (Fig. 5). These complexes are known to be among the most functionally essential and highly conserved proteins in the cell (31–34). The results suggest that maintenance of specific turnover rates may be critical to conservation of function in some proteins.

## DISCUSSION

It has long been known that within a cell, proteins have a range of turnover rates. However, the functional significance of a protein's half-life has been difficult to ascertain. The range of degradation rates within the cell are far narrower than other kinetic parameters that influence protein levels such as transcription rates, mRNA stabilities and translation rates, and as such, play a comparatively minor role in establishing the steady-state level of proteins (9). Furthermore, previous studies had found relatively low level of conservation in protein half-lives among species (8, 20, 21). However, these studies were based on comparisons of datasets collected from few organisms using varying methodologies, cell types or tissues. In this study, we provide a systematic comparison of proteome turnover rates within a single cell type across multiple species and assess their inter-species variability. The data shed light on the evolution and functional relevance of global protein turnover kinetics.

Our results indicate that turnover kinetics are highly correlated among closely related rodent species (less than 50 MYA divergence time). Indeed, for the most closely related pairs of species (*e.g.* rat and mouse) the variability in *k_degradation_* measurements are fully accounted for by experimental error alone. We further observed that *k_degradation_* correlations decrease as a function of evolutionary distance. However, this trend was not observed for the subset of proteins whose sequences were fully conserved among species, suggesting that divergence in relative turnover rates of proteins are related to changes in their amino acid sequence over the course of evolution.

The above result may be interpreted in two ways. It is known that specific sequence motifs act as a targeting signal for degradation. Additionally, changes in amino acid sequence may alter physical properties of proteins (*e.g.* thermodynamic stability or folding efficiency) that modulates the susceptibility of the protein to degradation. Thus, changes in sequences of target proteins may directly alter their turnover kinetics. Alternatively, it can be argued that proteins with conserved sequences are likely to be the most functionally critical proteins in the cell. As such, alterations in their turnover kinetics may be more consequential to viability. Thus, the selectivity of the degradation machinery in a cell may have evolved to maintain a specific degradation rate for these proteins during the course of evolution. In the former scenario, alterations in sequence directly cause changes in degradation rates, whereas in the latter scenario, conservation of sequence and turnover kinetics merely correlate with one another and are not causatively linked. Our current data cannot distinguish between these two possibilities.

In addition to differences in correlation between pairs of species, we observed systematic shifts in the distribution of *k_degradation_* values among some species. Notably, we observed a strong negative correlation between median *k_degradation_* values and maximal life spans of rodents, indicating that long-lived species had slower overall rates of degradation in comparison to short-lived species. This correlation was somewhat unexpected in light of numerous studies indicating that *faster* protein turnover is generally beneficial for healthy aging (35–37). Indeed, in recent years, decelerated protein turnover has been linked to the process of aging in several different contexts. For example, it has been observed that as organisms age, activities of the UPS and autophagy pathways decrease. This loss of “proteostasis” has been highlighted as one of the hallmarks of aging (38). Additionally, several age-related neurodegenerative disorders such as Parkinson's and Alzheimer's disease are caused by protein aggregation and the loss of proteostasis that results from the accumulation of stable aggregates in the cell. Also, several genes and pathways associated with longevity, including mTOR, Ins/IGF-1 and FoxO, are known to be upstream regulators of autophagy or UPS (39–41). Together, these observations have led to the idea that the maintenance of robust protein turnover is an important mechanism for avoiding detrimental aging phenotypes.

However, it is important to note that protein turnover is one of the most energetically demanding processes in living organisms. Protein synthesis and most protein degradation pathways require the hydrolysis of ATP. Indeed, constitutive protein turnover accounts for as much as 25% of the total energy expenditure in the body (42). Much of the ATP produced in the body is generated through the process of oxidative phosphorylation. However, a byproduct of oxidative phosphorylation is the stochastic generation of reactive oxygen species (ROS). It is well known that accumulation of ROS results in oxidative damage to a number of macromolecules including proteins, DNA and lipids and is associated with several aging phenotypes (43). Thus, although the replenishment of undamaged proteins by constitutive turnover is generally beneficial to the cell, its significant contribution to the generation of ROS may diminish its long-term benefit to the organism.

It is therefore possible that although enhanced constitutive protein turnover is a proteostatically favorable process in short-lived organisms, in long-lived organisms, where ROS damage can accumulate in post-mitotic tissues over an extended lifespan, its benefits are outweighed by its liabilities. Thus, instead of non-selective turnover of the bulk protein population, long-lived organisms may be more reliant on selective degradative mechanisms for specific detection and clearance of damaged proteins. Such a strategy would maintain the proteostatic benefits of protein degradation while minimizing its energetic demands. Alternatively, long-lived organisms may have evolved more robust quality control mechanisms in order to minimize the occurrence of protein damage and negate the proteostatic need for faster turnover rates. In support of this idea, it has been shown that longer lived rodents display a higher level of translational fidelity (44). Nonetheless, understanding the complete functional significance of slow turnover rates in long-lived organisms will require further study.

## Data Availability

All raw and processed data are available at ProteomeXchange Consortium via the PRIDE database (www.ebi.ac.uk/pride/; accession number PXD007598).

## Supplementary Material

Supplemental Data

## Footnotes

^1^ The abbreviations used are:

UPS: ubiquitin proteasome system
AGC: automatic gain control
CID: collision induced dissociation
SILAC: stable isotopic labeling in cell culture
CV: coefficient of variation
LC-MS/MS: liquid chromatography tandem mass spectrometry
PRIDE: proteomics identifications
r_s_: Spearman's rank correlation coefficient
r: Pearson's correlation coefficient
GO: gene ontology
mRNA: messenger ribonucleic acid
ROS: reactive oxygen species
DTT: dithiothreitol
FBS: fetal bovine serum
EMEM: Eagle's minimum essential medium
BCA: bicinchoninic assay
PBS: phosphate buffer saline
MEM: minimum essential medium
TCEP: Tris(2-carboxyethyl)phosphine
IAA: iodoacetamide
RT: room temperature
TFA: trifluoric acid
mTOR: mammalian target of rapamycin
IGF-1: insulin growth factor-1
ATP: adenosine triphosphate
DNA: deoxyribonucleic acid
REVIGO: reduce and visualize gene ontology
PSM: peptide spectral match.
