# Supplementary material for: Cross-species Comparison of Proteome Turnover Kinetics
Source: Mol Cell Proteomics. 2018 Jan 10;17(4):580–91. doi: 10.1074/mcp.RA117.000574 (PMC5880112; doi:10.1074/mcp.RA117.000574)
Supplement: Supplemental Data [file supp_17_4_580__index.html]

Cross-species comparison of proteome turnover kinetics — Cross-species comparison of proteome turnover — Cross-species Comparison of Proteome Turnover Kinetics — Cross-species Comparison of Proteome Turnover — Supplemental Data 

# Cross-species Comparison of Proteome Turnover Kinetics

## Supplemental Data

- Supplementary Information - Supplementary Figures
- Supplementary Tables - Supplementary Tables
- Supplementary Table S2 - Supplementary Table S2 (this file was uploaded separately due to its large size that exceeded the upload limit)
