# Supplementary material for: Cross-species Comparison of Proteome Turnover Kinetics
Source: Mol Cell Proteomics. 2018 Jan 10;17(4):580–91. doi: 10.1074/mcp.RA117.000574 (PMC5880112; doi:10.1074/mcp.RA117.000574)
Supplement: Supplemental Data [file supp_RA117.000574_134905_0_supp_43353_p1c56y.pdf]

ASSOCIATED CONTENT

Spectral search results, SILAC quantitations, and rate measurements at peptide and protein levels are provided in tabular format in the supplementary information.

Supplemental Figures

Figure S1. Variation of $k_{degradation}$ values for peptides matched to the same proteins

Figure S2. Correlation between maximal lifespan and median degradation rate after phylogenetic correction using the method of independent contrasts

Figure S3. Pairwise comparisons of protein $k_{degradation}$ values between species

Figure S4. Gene Ontology enrichment analysis of proteins with low $k_{degradation}$ CV values

Supplemental Tables

Table S1. MaxQuant search parameters (Table S1.xls)

Table S2. Peptide Level Search and Quantitation Information (Table S2.xls)

Table S3. Protein Level Search and Quantitation Information (Table S3.xls)

Table S4. Peptide-Level $k_{degradation}$ measurements (Table S4.xls)

Table S5. Protein-Level $k_{degradation}$ measurements (Table S5.xls)

Table S6. Conserved proteins between mouse, rat, hamster and guinea pig (Table S6.xls)

Table S7. Gene Ontology enrichment analysis of proteins with low $k_{degradation}$ CV values (Table S7.xls)

(Note: Detailed descriptions of Tables S1-S7 are contained within each file)

Accession Numbers

All raw and processed data are available at ProteomeXchange Consortium via the PRIDE database (accession number: PXD007598, username: reviewer91553@ebi.ac.uk, password: XEVQlPqK)

SUPPLEMENTARY FIGURE LEGENDS

Figure S1. Variance of peptide $k_{degradation}$ measurements. Box plots indicate the range of coefficient of variations (CVs) for measured fractional labeling of peptides mapped to the same protein. The dots indicate the CV for all peptides at a given time-point.

Figure S2. Correlation between maximal lifespan and median degradation rate after phylogenetic correction using the method of independent contrasts. Refer to (29) for detailed description of the statistical methodology.

Figure S3. Cross-species correlations of $k_{degradation}$ measurements. Pairwise associations of $k_{degradation}$ measurements between all analyzed species. The measured r_s values are plotted in Figure 5.

Figure S4. Gene Ontology (GO) terms enriched in proteins with low $k_{degradation}$ variance among eight species. The analysis was conducted using the algorithm GOrilla (30) for analysis of gene list ranked by CV and visualized by REVIGO (47). The sizes of the circles indicate the number of genes contained in the GO term, shading indicates the statistical significance of GO enrichment and gray lines indicate the member overlap between categories.

SUPPLEMENTARY FIGURES

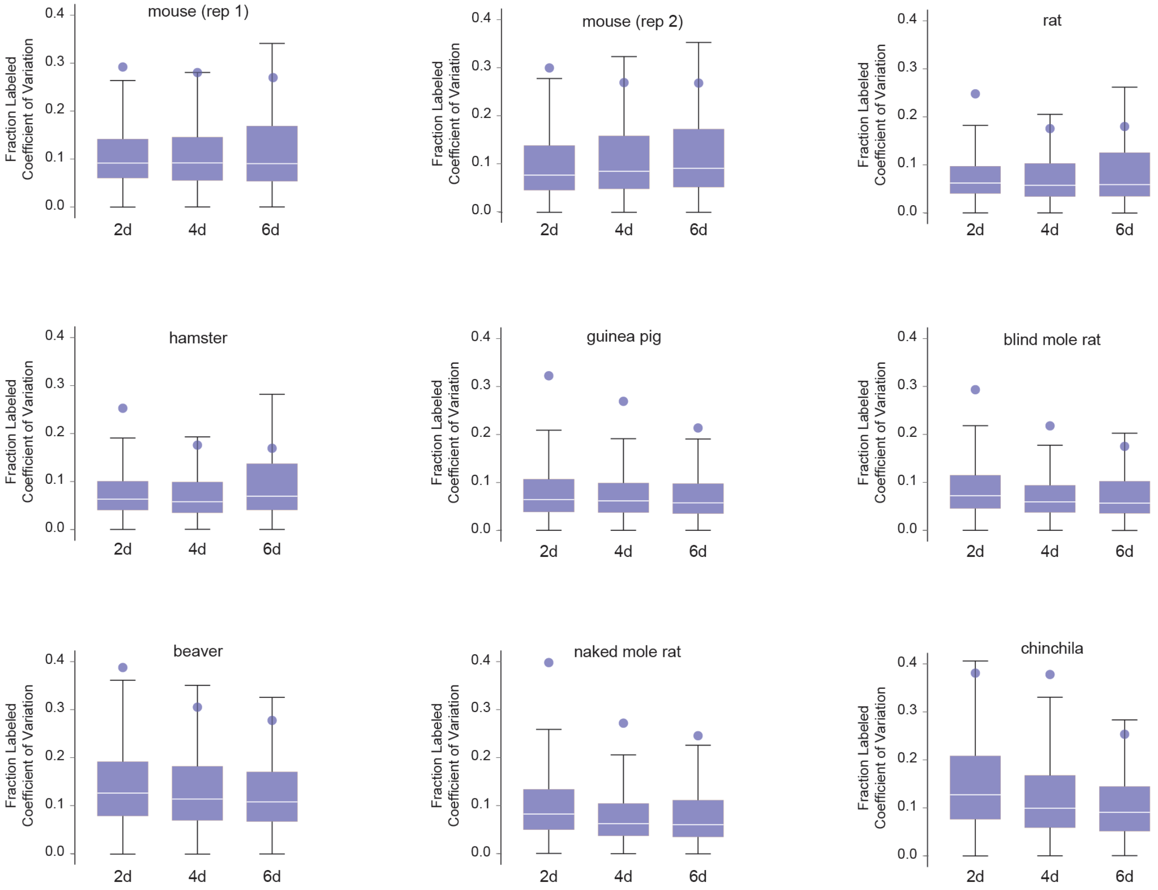
mouse (rep 1)
mouse (rep 2)
rat
hamster
guinea pig
blind mole rat
beaver
naked mole rat
chinchila
Fraction Labeled Coefficient of Variation
0.0
0.1
0.2
0.3
0.4
2d
4d
6d

Figure S1

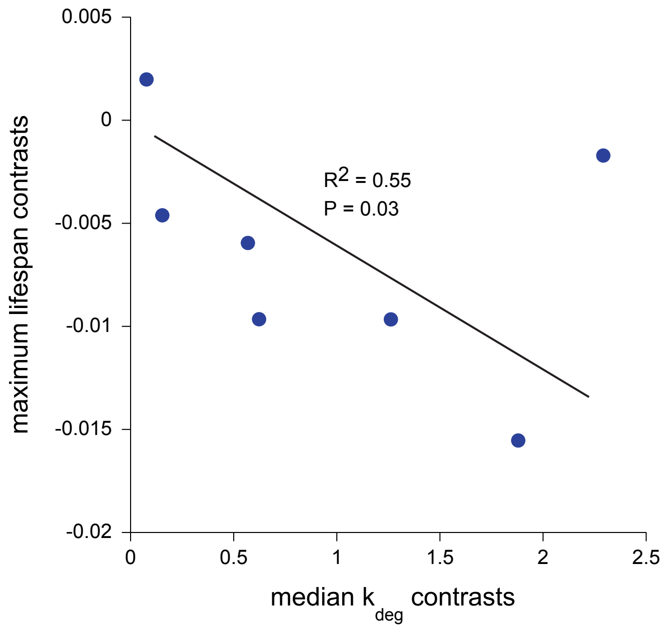
0.005
0
-0.005
-0.01
-0.015
-0.02
0
0.5
1
1.5
2
2.5
maximum lifespan contrasts
median k deg contrasts
R2 = 0.55
P = 0.03

Figure S2

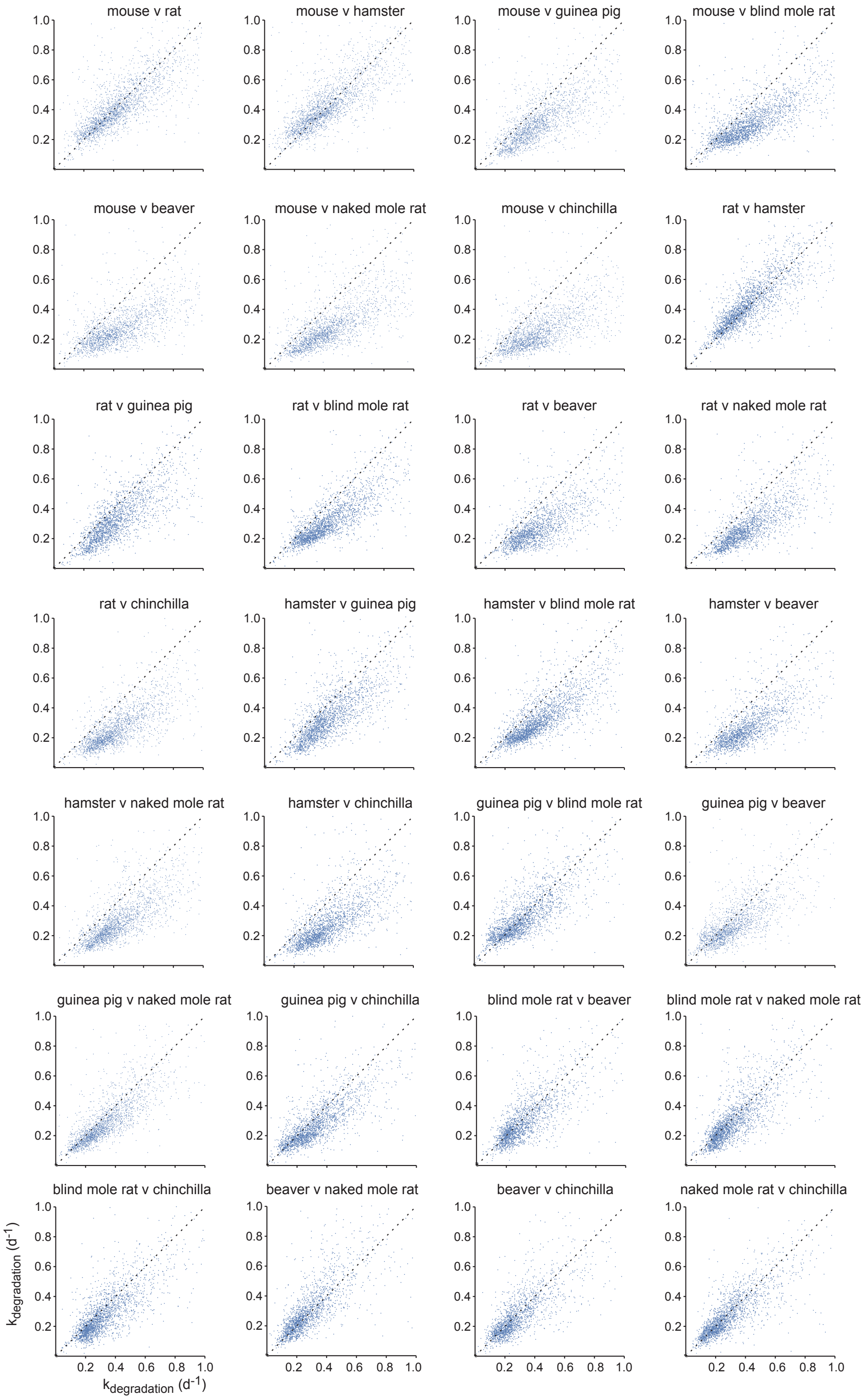
mouse v rat
mouse v hamster
mouse v guinea pig
mouse v blind mole rat
mouse v beaver
mouse v naked mole rat
mouse v chinchilla
rat v hamster
rat v guinea pig
rat v blind mole rat
rat v beaver
rat v naked mole rat
rat v chinchilla
hamster v guinea pig
hamster v blind mole rat
hamster v beaver
hamster v naked mole rat
hamster v chinchilla
guinea pig v blind mole rat
guinea pig v beaver
guinea pig v naked mole rat
guinea pig v chinchilla
blind mole rat v beaver
blind mole rat v naked mole rat
blind mole rat v chinchilla
beaver v naked mole rat
beaver v chinchilla
naked mole rat v chinchilla
$k_{degradation}$ (d^{-1})
$k_{degradation}$ (d^{-1})
0.2 0.4 0.6 0.8 1.0

Figure S3

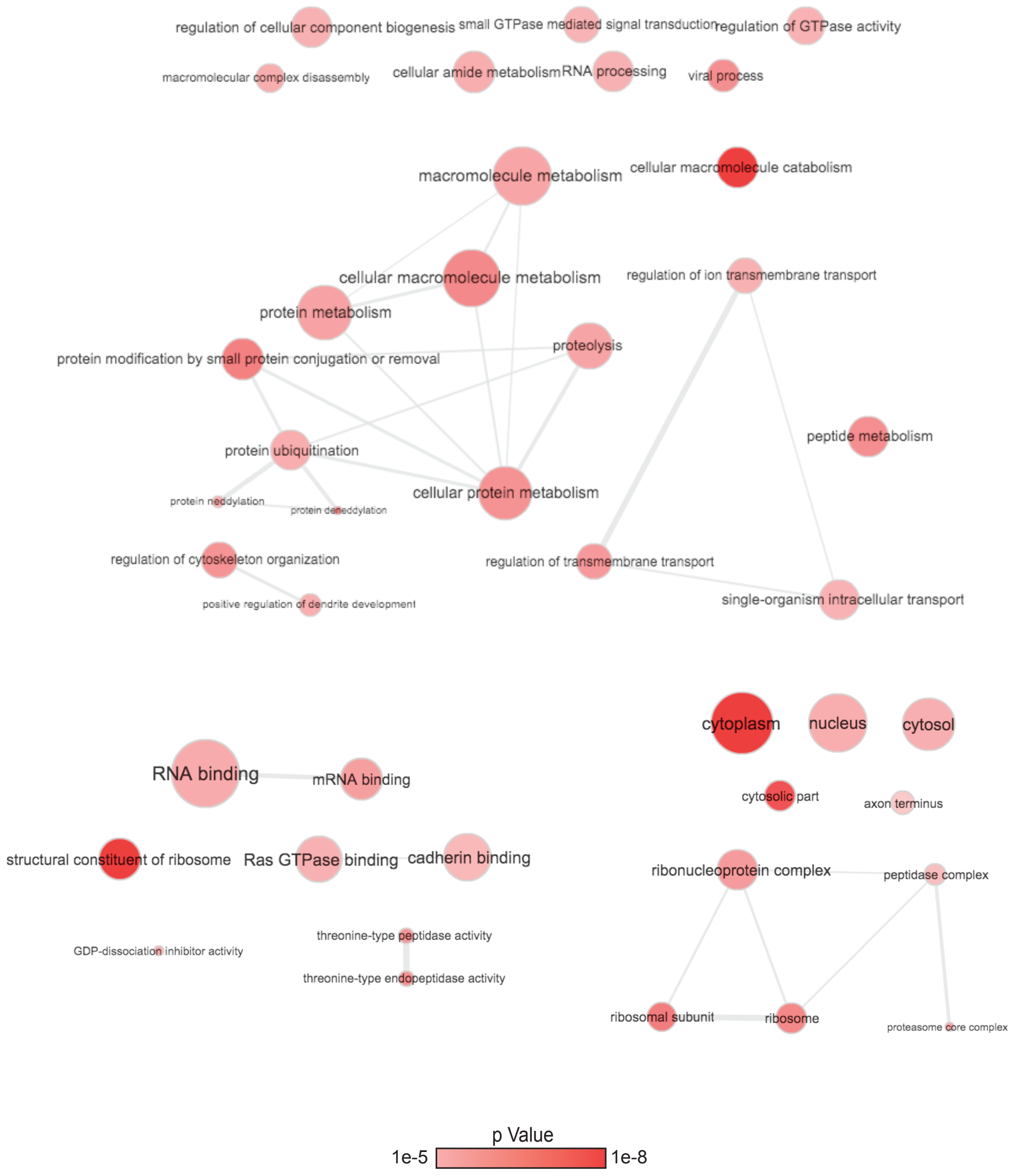
regulation of cellular component biogenesis
small GTPase mediated signal transduction
regulation of GTPase activity
macromolecular complex disassembly
cellular amide metabolism
RNA processing
viral process
macromolecule metabolism
cellular macromolecule catabolism
cellular macromolecule metabolism
regulation of ion transmembrane transport
protein metabolism
proteolysis
protein modification by small protein conjugation or removal
peptide metabolism
protein ubiquitination
protein neddylation
protein deneddylation
cellular protein metabolism
regulation of cytoskeleton organization
regulation of transmembrane transport
positive regulation of dendrite development
single-organism intracellular transport
cytoplasm
nucleus
cytosol
RNA binding
mRNA binding
cytosolic part
axon terminus
structural constituent of ribosome
Ras GTPase binding
cadherin binding
ribonucleoprotein complex
peptidase complex
threonine-type peptidase activity
GDP-dissociation inhibitor activity
threonine-type endopeptidase activity
ribosomal subunit
ribosome
proteasome core complex
p Value
1e-5
1e-8

Figure S4
